# Pre-Treatment of Platinum Resistant Ovarian Cancer Cells with an MMP-9/MMP-2 Inhibitor Prior to Cisplatin Enhances Cytotoxicity as Determined by High Content Screening

**DOI:** 10.3390/ijms14012085

**Published:** 2013-01-22

**Authors:** Alexandros Laios, Bashir M. Mohamed, Lynne Kelly, Richard Flavin, Stephen Finn, Lynda McEvoy, Michael Gallagher, Cara Martin, Orla Sheils, Martina Ring, Anthony Davies, Margaret Lawson, Noreen Gleeson, Tom D’Arcy, Charles d’Adhemar, Lucy Norris, Ream Langhe, Feras Abu Saadeh, John J. O’Leary, Sharon A. O’Toole

**Affiliations:** 1 Department of Obstetrics and Gynaecology, Trinity College Dublin, Trinity Centre for Health Sciences, St. James’s Hospital, Dublin 8, Ireland; E-Mails: alxlaios2000@yahoo.com (A.L.); lynne.k.la@gmail.com (L.K.); lmmcevoy@tcd.ie (L.M.E.); noreengleeson@dubgyn.org (N.G.); tdarcy@coombe.ie (T.D.A.); lnorris@tcd.ie (L.N.); langher@tcd.ie (R.L.); ferasabusaadeh@dubgyn.org (F.A.S.); 2 Department of Histopathology, Trinity College Dublin, Sir Patrick Duns Research Laboratory, St. James’s Hospital and The Coombe Women and Infants University Hospital, Dublin 8, Ireland; E-Mails: flavinrichard@gmail.com (R.F.); Stephen.finn@tcd.ie (S.F.); gallagmi@tcd.ie (M.G.); cmartin3@tcd.ie (C.M.); osheils@tcd.ie (O.S.); mring@coombe.ie (M.R.); cjdadhemar@gmail.com (C.D.A.); 3 Department of Clinical Medicine, Trinity College Dublin, Trinity Centre for Health Sciences, St. James’s Hospital, Dublin 8, Ireland; E-Mails: bashmohamed@gmail.com (B.M.M.); amitche@tcd.ie (A.D.); 4 Department of Histopathology, St. James’s Hospital, Dublin 8, Ireland; E-Mail: magzzy2001@hotmail.com

**Keywords:** ovarian, MMP-9/MMP-2 inhibitor, chemoresistance, recurrent, high content screening

## Abstract

Platinum resistance is a major cause of treatment failure in ovarian cancer. We previously identified matrix metalloproteinase 9 (MMP-9) as a potential therapeutic target of chemoresistant disease. A2780cis (cisplatin-resistant) and A2780 (cisplatin-sensitive) ovarian carcinoma cell lines were used. The cytotoxic effect of MMP-9/MMP-2 inhibitor, (2*R*)-2-[(4-Biphenylsulfonyl) amino]-3 phenylpropionic acid (C21H19NO4S) alone or in combination with cisplatin was determined using high content screening. Protein expression was examined using immunohistochemistry and ELISA. Co-incubation of cisplatin and an MMP-9/MMP-2 inhibitor, (2*R*)-2-[(4-Biphenylsulfonyl) amino]-3 phenylpropionic acid (C21H19NO4S) resulted in significantly greater cytotoxicity as compared to either treatment alone in a cisplatin resistant MMP-9 overexpressing cell line; A2780cis. In addition, pre-incubating with MMP-9i prior to cisplatin further enhances the cytotoxic effect. No significant difference was observed in MMP-9 protein in tissue but a trend towards increased MMP-9 was observed in recurrent serum. We propose that MMP-9/MMP-2i may be utilized in the treatment of recurrent/chemoresistant ovarian cancers that overexpress MMP-9 mRNA but its role *in vivo* remains to be evaluated.

## 1. Introduction

Platinum compounds, given as cis- or carboplatin, constitute the most active and standard chemotherapy treatment for nearly all women diagnosed with ovarian cancer. Despite its efficacy and resistance to chemotherapy, both intrinsic and acquired, it is a major cause of treatment failure. Resistance to cisplatin occurs in roughly one-third of women during primary treatment and in almost all patients treated for recurrent disease [1]. Currently, it can only be determined retrospectively after patients have experienced the burden and toxicity of ineffective therapy.

In an attempt to determine novel biomarkers in recurrent/chemoresistant ovarian cancer, we identified distinct patterns of gene expression between primary and recurrent ovarian cancers and we proposed an integrative mRNA and miRNA model of recurrence in ovarian cancer [2,3]. In these studies, matrix metalloproteinase MMP-9 was identified as a potential marker of recurrence/chemoresistance and miRNAs predicted to target this gene were dysregulated between primary and recurrent specimens. The current study extended this finding to examine the protein expression of MMP-9 in tissue and serum of primary and recurrent/chemoresistant ovarian cancers.

MMPs form a family of zinc dependent endopeptidases responsible for the proteolysis of extracellular matrix (ECM) components upon various biological processes including carcinogenesis, differentiation, apoptosis, migration and invasion, regulation of tumor angiogenesis and immune surveillance [4]. They have been primarily associated with matrix remodeling and degradation of ECM, essential components of tumor invasion and spread to local and distant sites. This is particularly important in ovarian cancer where invasion and metastasis remain the most important characteristics. They can also critically regulate the tumor microenvironment [5] and their expression is increased in almost all human cancers compared to normal tissue.

The two MMPs most closely correlated with metastatic potential are the 72 kDa MMP-2 (gelatinase A) and the 92 kDa MMP-9 (gelatinase B) that function by degrading type IV collagen present in the basement membrane. Metastatic tumor cell lines have higher levels of gelatinases than nonmetastatic counterparts [6].

Dysregulation of MMPs has been shown to be central to tumor progression [7]. MMP-9 and MMP-2 have been found consistently upregulated in ovarian cancer and are associated with poor prognosis [8–12]. In ovarian cancer, MMP-9 is expressed in both the stroma and the ovarian epithelial tumor cells and MMP-9 expression in either compartment is indicative of poor prognosis [13].

Clinical trials with broad-spectrum MMP inhibitors in cancer patients have shown dose limiting toxicities [14] and no therapeutic efficacy due in part, to their lack of specificity for tumor-associated MMPs. Most trials use the maximum tolerated dose of inhibitors and apart from the obvious adverse side-effects, some inhibitors lose selectivity for specific MMPs [15]. This necessitates either reduced dosing or highly selective targeting, or even use in earlier stage than conventional chemotherapeutics.

To investigate the role of MMP-9 in recurrent/chemoresistant ovarian cancer we carried out functional inhibition of the gene using a chemical inhibitor (2*R*)-2-[(4-Biphenylsulfonyl) amino]-3 phenylpropionic acid (C_21_H_19_NO_4_S) (MMP-9/MMP-2i). We examined the cytotoxic effect of the inhibitor alone or in conjunction with cisplatin. Cytotoxicity was evaluated using a High Content Screening (HCS) multiparametric cytotoxicity assay. Cell based HCS assays have shown high potential for predictive toxicity assessments, mainly because they can simultaneously measure several of the features involved in chemical and environmentally presented nanomaterials induced toxicity [16–19]. Furthermore, this technology is becoming an indispensable approach to research and industry, assisting in understanding complex cellular processes related to disease pathogenesis [20], drug target validation and lead to the drug identification [21,22].

## 2. Results and Discussion

### 2.1. Results

#### 2.1.1. RNA Sample Analysis from Cell Lines

**Figure 1:**
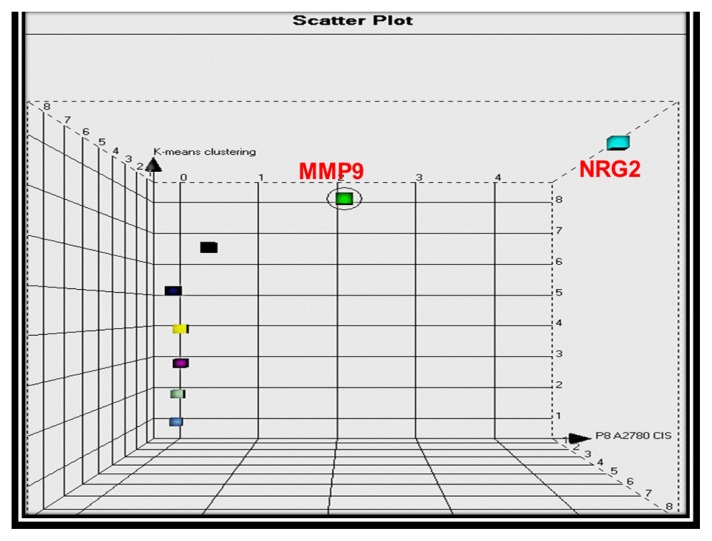
Scatter plot three dimensional clustering of tested genes based on their fold changes in A2780cis compared to A2780 cell lines. Each gene represents one point with three attributes (*x*: A2780cis; *y*: A2780; *z*: Hierarchical clustering) and is placed as a coordinate in an attribute space based on Euclidean distance measurements. The scatter plot demonstrated a main group of genes with similar fold changes. MMP9 and NRG2 appeared to “skew” from that main group given their upregulation pattern in A2780cis *vs.* A2780 cells with fold changes 2.12 and 4.37 respectively.

The quality and quantity of RNA extracted from A2780cis and A2780 ovarian cancer cells was determined prior to TaqMan analysis using a NanoDrop^®^ spectrophotometer (Thermo Fisher Scientific: Wilmington, DE, USA). TaqMan PCR was carried out on cell lines using previously tissue validated targets (*n* = 11). Relative quantitation was carried out in A2780cis *vs.* A2780 ovarian cancer cells over three passages. MMP-9 and NRG2 were identified as upregulated in “resistant” *vs.* “sensitive” cells with fold changes of 2.12 and 4.37 respectively (Figure 1). MMP-9 expression was further investigated in a different sensitive and resistant pair of ovarian cell lines; IGROV and IGROV_CDDP,_ but no increase was observed in the platinum resistant line of this pair. A chemical inhibitor was commercially available for MMP-9, so this was chosen for further analysis. MMP-9i is also an inhibitor of MMP-2, however, no significant difference was noted in MMP-2 expression and the MMP-9i used in this study is more selective for MMP-9. For abbreviations reasons, MMP-9i/MMP-2i will be mentioned as MMP-9i in the figures and tables below.

#### 2.1.2. Cisplatin Induces Cell Death in Cisplatin Resistant Ovarian Cancer Cells

**Figure 2:**
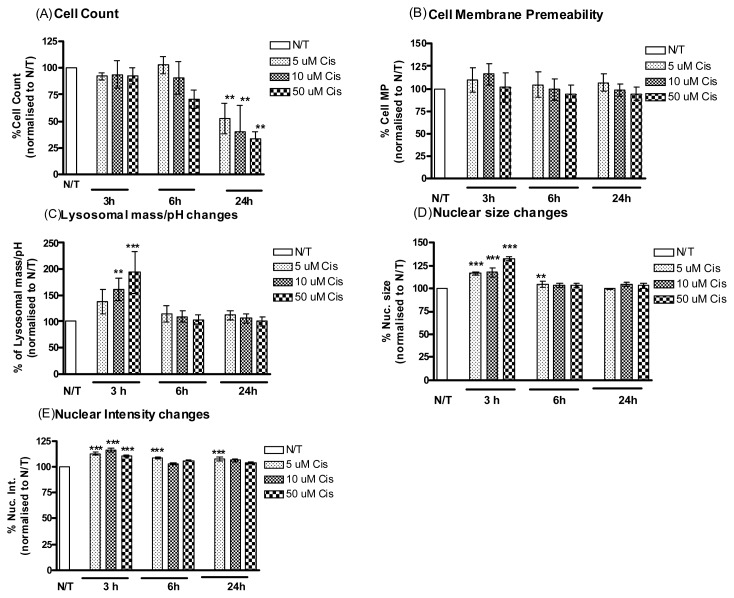
A2780cis cells treated with cisplatin. Simultaneous monitoring of changes in (**A**) cell count, (**B**) cell membrane permeability, (**C**) lysosomal mass/pH, (**D**) nuclear size and (**E**) nuclear condensation/intensity following treatment of the A2780cis cells with cisplatin (5 μM, 10 μM, 50 μM) and incubation for different time points 3, 6 and 24 h. Values were normalised to vehicle treated wells. Representative data are shown as means ±Standard Error (SE) (*n* = 3), **p < 0.01, ***p < 0.001.

A2780cis cells were assessed for cisplatin-induced cytotoxicity by incubating with 5, 10 and 50 μM of cisplatin for 3, 6 and 24 h time periods (Figure 1). Values were normalized to untreated controls. A significant decrease was observed in cell viability after 24 h incubation with the most significant decrease seen with the highest concentration of cisplatin (Table 1, upper panel). The cells were assessed for 4 cytotoxicity parameters, cell membrane permeability, lysosomal mass, nuclear size and nuclear intensity. A significant cytotoxic effect was observed after 3 h incubation as indicated by the measurements of lysosomal mass/pH, nuclear morphology changes. The peak plasma concentration (PPC) of cisplatin (5 μM)—and not a higher concentration—was sufficient to cause a significant increase in nuclear size and intensity at 6 h and in nuclear intensity at 24 h (Figure 2C–E).

#### 2.1.3. MMP-9/MMP-2i Induces Cell Death in Cisplatin Resistant Ovarian Cancer Cells

**Figure 3:**
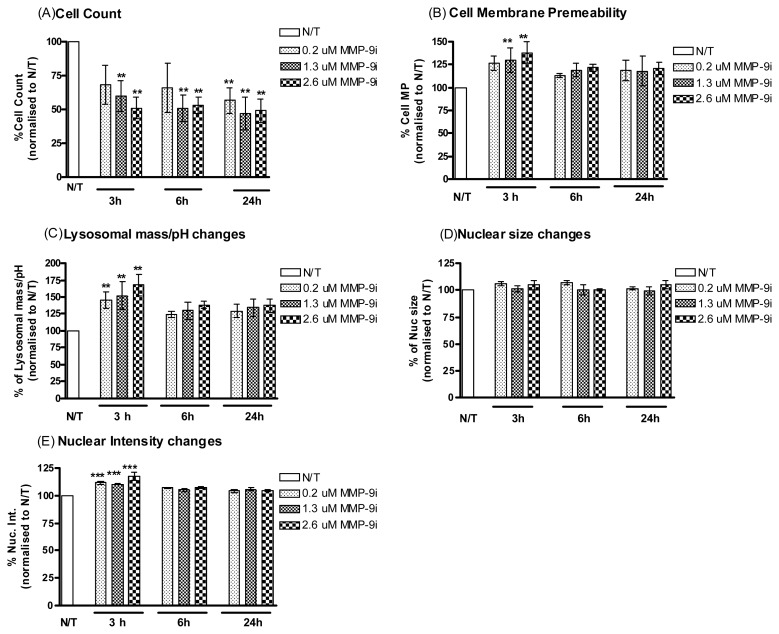
A2780cis cells treated with MMP-9/MMP-2i. Simultaneous monitoring of changes in (**A**) cell count, (**B**) cell membrane permeability, (**C**) lysosomal mass/pH, (**D**) nuclear size and (**E**) nuclear condensation/intensity following treatment of the A2780cis cells with MMP-9/MMP-2i (0.2 μM, 1.3 μM, 2.6 μM) and incubation for different time points 3, 6 and 24 h. Values were normalized to vehicle treated wells. Representative data are shown as means ± SE (*n* = 3), **p < 0.01, ***p < 0.001.

To determine if MMP-9/MMP-2i alone was cytotoxic to resistant ovarian cells *in vitro*, cells were treated with or without MMP-9/MMP-2i at titrated concentrations (0.2 μM, 1.3 μM, 2.6 μM) for 3, 6 and 24 h (Figure 3). MMP-9/MMP-2i caused a significant reduction in cell viability in a dose- and time-dependent manner, which was more significant than cisplatin alone treatment (Table 1, upper panel). Significant increases were observed at the early time point of 3 h for cell membrane permeability, lysosomal mass and nuclear intensity, which are indicative of a cytotoxic effect (Figure 3B,C,E).

#### 2.1.4. MMP-9/MMP-2i Enhances Cisplatin-Induced Cell Death in Chemoresistant Ovarian Cancer Cells at an Early Time Point

**Figure 4:**
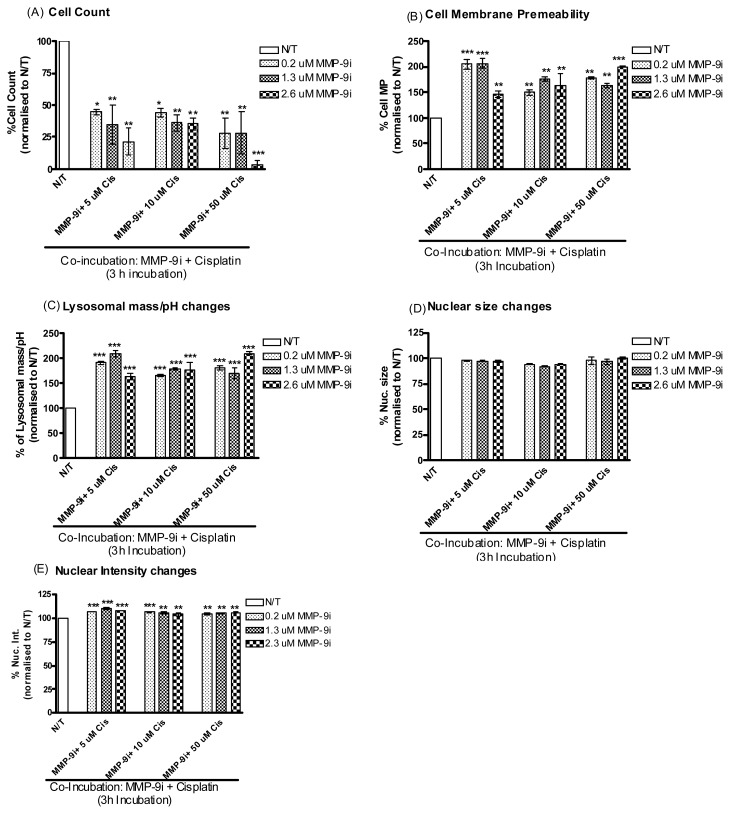
Co-incubation of A2780cis cells with cisplatin and MMP-9/MMP-2i. simultaneous monitoring of changes in (**A**) cell density, (**B**) cell membrane permeability, (**C**) lysosomal mass/pH, (**D**) nuclear condensation/intensity and (**E**) nuclear size following co-incubation of A2780cis cells with cisplatin and MMP-9/MMP-2i for 3 h. Values were normalized to vehicle treated wells. Representative data are shown as means ± SE (*n* = 3), *p < 0.05, **p < 0.01, ***p < 0.001.

We then sought to determine whether co-treatment of ovarian cancer cells with MMP-9/MMP-2i could enhance cisplatin-induced cytotoxicity at the early time point of 3 and 6 h. Resistant ovarian cancer cells were co-incubated with cisplatin and varying concentrations of MMP-9/MMP-2i (0.2–2.6 μm) (Figure 4). A significant decrease was observed in cell viability following co-incubation of MMP-9/MMP-2i and cisplatin, which was more effective than treating with either agent alone (Table 1, lower panel). This effect was observed at a 3 h timepoint for all concentrations. Significant cytotoxic effects were observed for cell membrane permeability, lysosomal mass and nuclear intensity for all concentrations at the 3 h timepoint. The lower concentrations of cisplatin and MMP-9i displayed the most cytotoxic effects with relative fluorescence values doubling for the cell membrane permeability and lysosomal mass parameters (Figure 4B,C). A lesser effect was observed after 6 h for these 4 parameters (Figure S1).

#### 2.1.5. Pre-Incubation with MMP-9/MMP-2i further Enhanced Cisplatin Induced Cytotoxicity

**Figure 5:**
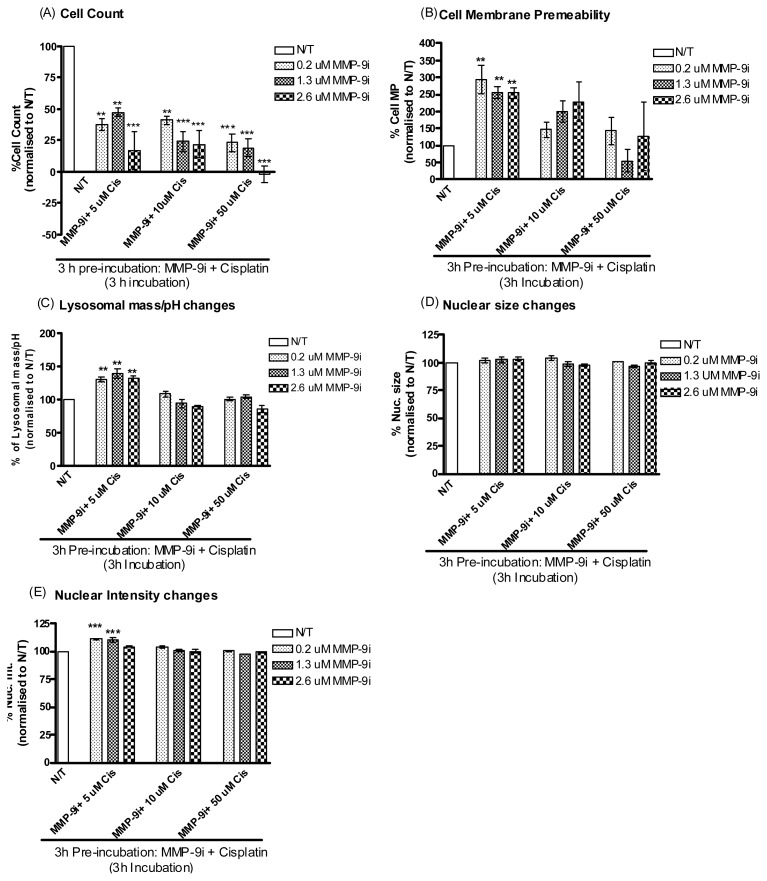
Pre-incubation of A2780cis cells with MMP-9/MMP-2i for 3 h followed by cisplatin treatment. Simultaneous monitoring of changes in (**A**) cell density, (**B**) cell membrane permeability, (**C**) lysosomal mass/pH, (**D**) nuclear condensation/intensity and (**E**). Nuclear size following pre-incubation of A2780cis cells with MMP-9/MMP-2i for 3 h followed by treatment with cisplatin for 3 h. Values were normalized to vehicle treated wells. Representative data are shown as means ± SE (*n* = 3), **p < 0.01, ***p < 0.001.

Further it was decided to investigate whether pre-incubating resistant ovarian cancer cells with MMP-9/MMP-2i for 3 h would result in more enhanced cytotoxicity. Indeed, pre-incubation with MMP-9/MMP-2i directly followed by treatment with cisplatin was significantly cytotoxic at the 3 h time point where a significant decrease in cell count was observed at all concentrations (Figure 5A and Table 1, lower panel). This cytotoxic effect was seen at the lower concentration of cisplatin in the cell membrane, lysosomal mass and nuclear intensity parameters at 3 h and it also followed through to the 6 h incubation period (Figure 5B,C,E and Figure S2).

#### 2.1.6. MMP-9/MMP-2i and Cisplatin Induces Cell Death in Chemosensitive Ovarian Cancer Cells

As a control, the same treatment regimens were performed (Figures S3–S6) on the A2780 sensitive cells, which do not overexpress MMP-9. The cytotoxic effects were much less than seen with A2780cis and at times only observed for the cell viability parameter. However, cytotoxicity was enhanced when pre-incubation with MMP-9/MMP-2i was performed (Figures S3–S6).

#### 2.1.7. Evaluation of MMP-9 Expression by Immunostaining *in vivo*

No significant difference of MMP-9 protein was observed between primary and recurrent/chemoresistant ovarian cancers. A subset of 6 primary and recurrent/chemoresistant tissues from the same patients were also investigated and while one case demonstrated marginal increased staining in the recurrent specimen this was not observed in the other cases where both primary and recurrent/chemoresistant expressed strong MMP-9 staining. An example of MMP-9 staining in a recurrent serous papillary adenocarcinoma of the ovary is shown in Figure S7.

#### 2.1.8. ELISA Analysis of Serum

A trend towards increased expression of MMP-9 was seen in recurrent/chemoresistant ovarian cancers with the reciprocal relationship observed in TIMP-2 levels (Figure S8). The study was confined to serous papillary adenocarcinomas and was limited by sample availability of recurrent serum and warrants investigation in a larger cohort.

### 2.2. Discussion

**Figure 6:**
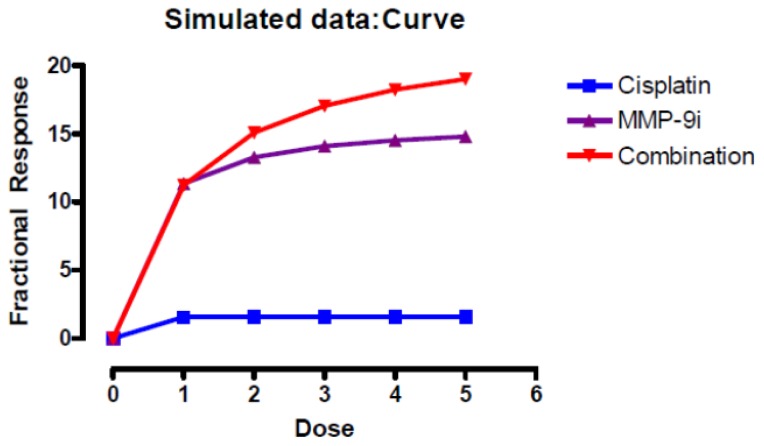
Synergy analysis of the MMP9/MMP2i with cisplatin and combination in the A2780cis cell line. The median-effect function of Chou and Talalay, assuming mutual exclusivity, using the CombiTool (version 2.001; IMB Jena Biocomputing Group: Jena, Germany) was applied to analyze the Bliss independence reference model. Plots show the different combination indices at various effect levels (fraction affected) for an experimental design in which the doses of cisplatin varied in the presence of a fixed dose of MMP-9/MMP2i. Values are means for 3 experiments (*n* = 4 for the 3 h experiment). The coefficient of variation for each set of experiments was <10%. An additive effect has been observed.

This study demonstrates the therapeutic potential of an MMP-9/MMP-2i inhibitor in combination with cisplatin for the treatment of recurrent/chemoresistant ovarian cancers overexpressing the MMP-9 gene. Using the novel high content screening technology we document for the first time an additive cytotoxic effect when MMP-9/MMP-2i is combined with cisplatin (Figure 6).

In addition, pretreating cisplatin resistant ovarian cancer cells with an MMP-9/MMP-2i inhibitor prior to treatment with cisplatin resulted in enhanced cytotoxicity at an early point. A recent study has demonstrated that MMP-9 siRNA significantly reduces the invasion and adhesive ability of ovarian cancer cells [23].

We previously identified the MMP-9 gene as upregulated in recurrent ovarian cancer tissue specimens [2]. We also identified a link between this gene and dysregulated miRNAs. The list of predicted mRNA targets for these miRNAs shared common significant pathways including actin and integrin signaling [3]. This is consistent with recent studies that directly relate MMP overexpression/ovarian cancer cisplatin resistance to actin cytoskeleton and integrins [24,25]. Other genes linked to MMP-9 such as the tissue inhibitors of matrix metalloproteinases (TIMPs) and zinc metabolism genes were also dysregulated in this cohort suggesting this gene may play an important role in recurrent/chemoresistant ovarian cancer. This difference was not observed at the protein level when MMP-9 was investigated by immunohistochemistry as both primary and recurrent/chemoresistant cases exhibited strong staining and subtle changes are difficult to interpret by immunohistochemistry. We also used a previously available TMA of primary and recurrent serous ovarian specimens. These were not all platinum-sensitive or platinum-resistant to perfectly mimic the *in vitro* model, hence no significant difference was observed in the expression of MMP9 levels in the recurrent compared to the primary specimens. This discrepancy is possible as stromal cells also secrete MMP9—which may modulate the invasiveness of ovarian cancer cells—and may exert varying degrees of activity following chemotherapy [13].

Serum MMP-9 showed a pattern of increasing concentration across benign, borderline, malignant and recurrent/chemoresistant serous tumors and one of its inhibitors, TIMP-2 displayed the reciprocal. While this did not reach significance, it does warrant further investigation in a larger cohort. Recent studies have demonstrated the clinical utility of MMP-9 to discriminate between malignant and benign ovarian cancers, however its utility in the recurrent setting has not been investigated [23,26,27].

We observed consistently higher MMP-9 expression in a cisplatin resistant (A2780cis) ovarian cell line compared to its sensitive counterpart (A2780). This was maintained for successive passages and before treatment of the A2780cis cell line with cisplatin to maintain chemoresistance. Over expression of MMP-9 was not observed in the IGROV/IGROV_CDDP_ paired sensitive/resistant cell lines which is not surprising due to the multifactorial nature of platinum resistance; hence emphasising the need for individual testing of tumors for specific targeted therapy.

In recent years, much attention has been focused on the function of MMP enzymes, given their sophisticated pattern of modulating opposite effects in cancer progression. Their overexpression correlates with increased invasion, metastasis and shortened survival [28]. Their antimetastatic propensity was further investigated by the development of MMP inhibitors (MMPIs) as a new class of anticancer drugs. Despite extensive research, very few inhibitors specific for the gelatinases have been described to date; the most potent also inhibit several other MMP family members. Unfortunately, these MMP intervention strategies have met with limited clinical success and severe toxicities. The mechanism of these toxicities is widely assumed to be due to the poor selectivity of these compounds but this has not been confirmed. In addition, it is now recognized that among MMPs, some possess cancer-promoting activities while others tumor-inhibiting functions underlining the risk of using broad-spectrum MMPIs [29]. A recent study in colorectal cancer has suggested that selective targeting of the tumor rather than stromal cell MMP-9 would be an ideal candidate for antimetastatic therapy [30].

The exact mechanism of their action is not yet completely understood. Apoptosis is induced by many but not all endogenous inhibitors and not necessarily by synthetic MMP inhibitors, thus suggesting that the mechanism may not rely on the inhibition of a MMP [31]. Synthetic MMP inhibitors appear to mimic most of the actions of TIMPs including MMP inhibition but also cell growth and proliferation [32,33]. Our results impose more than one mechanism is taking place as we observe inhibition when MMP-9 is overexpressed but we also observe some cytotoxicity in the cell line that doesn’t overexpress MMP-9 but this effect was not as significant when the cell line was treated with MMP-9i alone. It may well be that the chemotherapy is responsible for the increased expression of MMP-9 as established by a recent study [34]. Further work performing whole genome transriptome array analysis is necessary to evaluate the mechanism of action of this inhibitor.

We used a novel gelatinase inhibitor, derived from N-sulfony-lamino acid, which appears to specifically inhibit MMP-9 and to a lesser extent MMP-2 [35]. Inhibition of gelatinase activity in implanted tumor tissues has been demonstrated by means of film *in situ* zymography [36]. Such inhibitors selectively target tumors because their overexpression makes them accessible for homing in the tumor vasculature. The cytotoxic effects we observed were often at the lower concentration of the inhibitor where it has a higher specificity for MMP-9. The inhibitor used in this study has been shown to possess a novel proapoptotic function when combined with TNFa, TRAIL or FAS ligands [37]. This alternative mechanism could explain the enhanced apoptosis observed between our inhibitor and cisplatin, although the proposed antineoplastic mechanism has not been tested in an ovarian cancer model. A novel inhibitor of MMP-2 and MMP-9, BAY 12-9566 appears to have a more favorable toxicity profile by perturbation of the cell cycle [38]. Oral treatment with the highly selective MMPI RO 28-2653 decreases the incidence of liver metastases due to reduced MMP-2 and MMP-9 concentration in pancreatic cancer models [39].

We implemented a live-cell multiparametric HCS cytotoxicity assay measuring cell density, nuclear morphology, lysosomal mass and cell membrane integrity; the principle parameters of compromised cell health. This parallel analysis of multiple markers for cytotoxicity allows early reversible and late irreversible effects to be distinguished, therefore it provides a more sensitive interpretation of compound-induced toxicity, offering increased specificity and selectivity for toxic events [40]. We tested critical early measurements of toxicity as assays that target late events in the process of cell injury are more likely to miss toxicities that require chronic exposure or exert adverse but not lethal effects. Preincubating cells with MMP-9i at all doses was significantly cytotoxic at 3 h suggesting an early apoptotic response. Some of the pre-incubation effects followed through to the 6 h incubation and this was probably due to the significant effects observed at 3 h which the cells are trying to adapt to, suggesting it may require continuous or a more extended course of treatment to completely arrest tumor growth. A similar effect was observed using this inhibitor for treatment of melanoma [37]. Our time course results support the notion that if MMP-9 is considered a chemoresistant marker, the ability to acquire drug resistance arises early during the tumorigenesis process, as recently proposed and validated in an ovarian cancer model [41] and is associated with immediate adaptive response to treatment.

## 3. Experimental Section

### 3.1. Cell Culture

Matched sensitive and cisplatin resistant cell lines were investigated for this study. Human ovarian epithelial carcinoma cell lines A2780 and A2780cis (the cisplatin resistant counterpart) were purchased from the European Collection of Cell Cultures (ECACC: Salisbury, UK) and cultured in RPMI-1640 medium supplemented with 10% fetal calf serum, l-glutamine, penicillin and streptomycin (Sigma: Steinheim, Germany) in a humidified 5% CO_2_, 95% air atmosphere. IGROV and IGROV_CDDP_, were cultured in RPMI supplemented with 10% fetal calf serum.

### 3.2. Preparation of Cell Lines for RNA Extraction

Following trypsinisation of cultured cells, between 1 and 5 million cells were centrifuged for 12 min at 1200 rpm and supernatant was removed. Cells were lysed using Buffer RLT (Qiagen). Three different passages were used. RNA extraction was performed using the RNeasy^®^ mini kit (Qiagen: West Sussex, UK) as per manufacturer’s instructions. RNA was quantified using the NanoDrop^®^ spectrophotometer.

### 3.3. TaqMan PCR Analysis of Selected Candidates

Eleven genes (CLDN16, S100B, BTC, IL27RA, FGF2, NRG2, S100A8, ZNF218, MMP9, TJP3, IL1R2) previously validated as upregulated in recurrent compared to primary ovarian cancers [2] were selected for TaqMan PCR analysis in the cell lines. All reactions were carried out on the ABI Prism 7000 Sequence detection system (Applied Biosystems, Applera UK: Cheshire, UK) using the TaqMan^®^ Universal PCR master Mix and TaqMan^®^ Gene Expression Assays (Applied Biosystems). Relative quantitation in A2780cis compared to A2780 cells was carried out using the delta delta cycle time (ΔΔCt) method with 18S ribosomal RNA as an endogenous control. Upregulation was observed for an average of three passages in A2780cis compared to A2780 cell line. Technical replicates were performed (*n* = 3) for a representative passage for each cell line.

### 3.4. Chemicals

MMP-9/MMP-2 inhibitor, (2*R*)-2-[(4-Biphenylylsulfonyl)amino]-3 phenylpropionic acid (C_21_H_19_NO_4_S), was purchased from Calbiochem (Merck, Darmstadt, Germany). It is described as a potent inhibitor of MMP-2 (IC_50_ = 310 nM) and MMP-9 (IC_50_ = 240 nM) [35]. Stock solutions of MMP-9i which correspond to the IC50 (1×, 0.2 μM) and a 5× (1.3 μM) and 10× (2.6 μM) concentration were made in DMSO. Cisplatin was obtained from Sigma-Aldrich (Sigma-Aldrich, Ireland Ltd.: Arklow, Ireland, IMB Jena Biocomputing Group, http://www.imb-jena.de). A 10 μM stock solution was prepared in Phosphate Buffer Saline (PBS).

### 3.5. Experimental Design

The peak plasma concentration of cisplatin (5 μM) was assessed in addition to a 2× (10 μM) and a 10× (50 μM) concentration. The toxic effect of MMP-9/MMP-2i and cisplatin alone or in combination was determined using the multiparameter cytotoxicity 1 kit. Cells were plated (8 × 10^3^) in triplicate in 96-well plates overnight and then treated with titrated concentrations of drugs for 3, 6 and 24 h. Only the inner 60 wells of 96-well plates were used due to evaporation-related edge effects in the outside wells. A pre-incubation for 3 h with MMP-9i prior to treatment with cisplatin as well as co-incubation at 3 and 6 h were assessed. DMSO was used as a vehicle control.

### 3.6. Multiparameter Cytotoxicity Assay Using HCS System

A multiparametric cytotoxicity assay was performed using Cellomics^®^ HCS reagent HitKit™ as per the manufacturer’s instructions (Thermo Fisher Scientific Inc.: Pittsburgh, PA, USA). This kit enables measurements of cell viability, cell membrane permeability and lysosomal mass/pH, which are toxicity-linked cellular markers. Following a toxic compound or nanomaterial insult, cells may respond with changes in nuclear size and/or morphology depending on the cell type and the compound as well as loss of cell membrane integrity [17,18].

Prior to staining, cells were washed with PBS, then 65 μL of a fluorophore mixture (supplied by the manufacturer) containing Hoechst 33342, lysosomal mass/pH indicator and cell membrane permeability dye were made up in 10% supplemented RPMI media. Cells were incubated with 50 μL of the fluorophore mixture for 40 min under tissue culture conditions to enable measurement of cytotoxicity indicators. Dead cells were washed away twice with 100 μL of PBS and 200 μL of wash buffer was added to each well. The experimental layout for the automated microscopic analysis, based on the In Cell analyzer 1000 (GE Healthcare: Uppsala, Sweden), was composed of untreated, MMP-9/MMP-2i and DMSO treated plates. All these were scanned and acquired in a stereology configuration of 6 randomly selected fields. Images were acquired in a stereology configuration of five randomly selected fields at 10× magnification using three detection channels with different excitation filters. The rate of cell viability and proliferation were assessed by the automated analysis of the nuclear count and morphology (DAPI filter); in parallel the fluorescent staining intensities reflecting cell permeability (FITC filter) and lysosomal mass/pH changes (TRITC filter) were also quantified for each individual cell present in the examined microscopic fields (IN Cell Investigator, GE Healthcare: Buckinghamshire, UK).

### 3.7. Immunohistochemical Staining and Analysis

Immunohistochemical investigation for MMP-9 expression was conducted on 5 μm primary and recurrent/chemoresistant ovarian sections using the avidin-biotin-peroxidase complex detection procedure as previously described [34]. A primary TMA of 20 serous papillary adenocarcinomas was used and 20 recurrent/chemoresistant ovarian cancers of mixed histology were examined. A subset of matched primary and recurrent/chemoresistant specimens from the same patient were also investigated. Stained tissue sections were blindly investigated by a histopathologist using a 1 (weak), 2 (moderate) and 3 (strong) scoring system.

### 3.8. Patient Serum Samples

Serum samples from 39 patients were assayed in this study; 10 benign serous cystadenomas, 10 borderline serous cystadenomas, 10 advanced malignant serous papillary adenocarcinomas and 9 recurrent/chemoresistant serous adenocarcinomas. Pre-operative blood samples were taken from patients undergoing surgery for ovarian disease in St James’s Hospital Dublin. This research project had approval of the hospital ethics committee and informed consent was obtained.

### 3.9. ELISA for MMP-9 and Its Inhibitor TIMP-2

The Quantikine TIMP-2 and MMP-9 Immunoassay kits (Catalog No. DTM200 and DMP900 respectively) from R&D systems were used. The ELISA was performed according to manufacturer’s protocols. Serum samples were diluted 1:50 prior to assay.

### 3.10. Statistical Analysis

For cell culture, all treated cells were assayed in triplicate and results were expressed as mean percent surviving cells ± SE. Statistical analysis was carried out by Student’s *t*-test or two-way ANOVA was carried out using the PRISM^®^ software. Statistical significance was inferred at *p* < 0.05. ELISA results were analyzed using SPSS for Windows, Release 18.0.1. 2001. Chicago: SPSS Inc.

An experimental design for comparison of a single anticancer drug dose response relation with that of the same anticancer drug in combination with a fixed concentration of MMP-9/MMP2i was used and the measured responses compared to the Bliss independence reference model of synergy using the CombiTool computer program (version 2.001, IMB Jena Biocomputing Group: Jena, Germany). The drug interaction index (I*x*) was calculated according to the method of Chou and Talalay [42] using CombiTool. I*x* values are geometric means ± SEM in the presence of a fixed concentration of MMP-9/MMP2i, I*x* < 1 = synergy; I*x* = 1 additivity and I*x* > 1 antagonism. The Bliss independence model is defined by the equation: E*xy* = E*x* + E*y* − E*x*E*y* for 0 < E < 1 and where E*xy* is the additive effect of drugs *x* and *y* as predicted by their individual effects E*x* and E*y*. In this case, E*xy* would be the effect (fractional survival) of the drug used in combination with cisplatin, and E*x* and E*y* the fractional survival of cells exposed to the drug alone and to the drug in combination with cisplatin, respectively [43]. Effective inhibitory concentrations (EC50) are best-fit values obtained from non-linear regression analysis of the sigmoidal dose-response relation for each drug alone or in combination with MMP9i/MMP2i. The potency ratio and associated 95% CI (confidence interval) were computed by subtracting the log EC50 of MMP9/MMP2i in combination with cispaltin from the log EC50 of drug alone and back-transformation (antilogarithm) of data. GraphPad QuickCalcs, Graphpad Prism (Version 4.03, GraphPad Software: San Diego, CA, USA) were used for data and graphic analysis.

## 4. Conclusions

**Figure 7:**
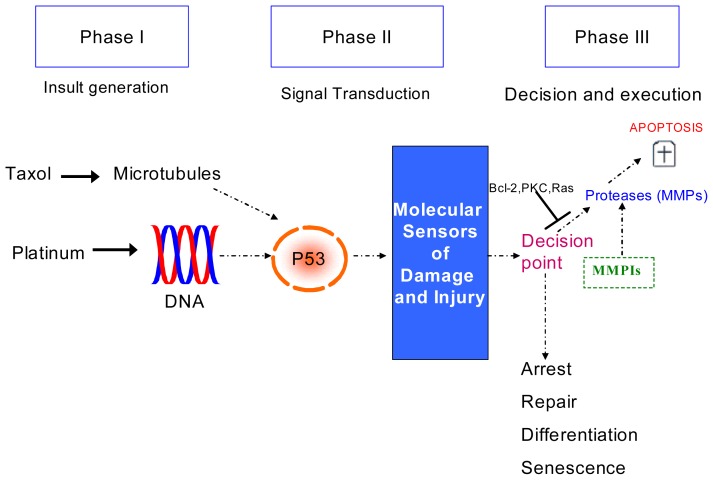
A model of possible implication of MMPIs in the treatment of ovarian cancer. Hypothesized phases in the induction of apoptosis in response to common chemotherapeutic agents in ovarian cancer. In phase I, cytotoxic agents impart damage to a critical component of the cell such as DNA or microtubules. In phase II, the cell recognizes the damage through signaling mechanisms. In phase III, the cell assesses the extent of damage and decides the appropriate response. In most cancer cells, the preferred response is the induction of apoptosis, whereas the response can also involve growth arrest to allow for repair or senescence or terminal cell differentiation. Cancer cells may acquire resistance to apoptosis at several points in this pathway. MMPs appear to induce the irreversible phase of apoptosis. Highly selective MMPIs may have a complementary role in the treatment of ovarian cancer and in particular in patients whose tumor overexpress MMP-9.

We report a successful adaptation of HCS for cytotoxicity using a novel MMP-9/MMP-2i alone and in combination with cisplatin and we provide evidence regarding highly selective MMP-9/MMP-2i involvement in the treatment of ovarian cancer (Figure 7). An enhanced early time and concentration-dependent cytotoxic effect was observed, consistent with a hormetic effect. This suggests that drug exposure may not have to be long-term and/or systematic thus reducing the possibility of side-effects. MMP-9 may have an important role in recurrent/chemoresistant cancer and further investigation of serum MMP-9 is warranted. This preliminary data suggests patients over expressing MMP-9 may benefit from the inclusion of an MMP-9 inhibitor in combination with chemotherapy. Our results suggest pretreating the cells with an MMP-9i before chemotherapy enhances the cytotoxic effect. Manipulation of doses, investigation of sub-toxic doses and use of antisense molecules to create highly selective MMPIs require further investigation.

## Supplementary Information



## Figures and Tables

**Table 1:** Tables showing percentage cell loss (%) following incubation with cisplatin and MMP-9/MMP-2i when A2780cis cells were treated with cisplatin alone, MMP-9/MMP-2i alone, cisplatin and MMP-9/MMP-2i (after 3 h incubation) and pretreatment of MMP-9/MMP-2i for 3 h prior to cisplatin treatment. Percentage cell loss (%) is normalized to vehicle treated cells (100%).

Time	Cisplatin concentration μM			MMP-9/MMP-2i concentration μM		

	5	10	50	0.2	1.3	2.6
3	8	6	8	32	40 **	49 **
6	+3	9	30	34	49 **	47 **
24	47 **	60 **	66 **	44 **	53 **	51 **

**Conc cis μM**	**Co-incubation 3 h** **Conc MMP-9/MMP-2i μM**			**Pre-incubation 3 h** **Conc MMP-9/MMP-2i μM**		

	**2**	**1.3**	**2.6**	**0.2**	**1.3**	**2.6**

5	55 ^*^	65 **	79 **	62 **	53 **	83 ***
10	56 ^*^	44 **	65 **	59 **	76 ***	78 ***
50	72 **	72 **	97 ***	77 ***	81 ***	100 ***

** *p* < 0.01,

*** *p* < 0.001.
