# Supplementary material for: Pre-Treatment of Platinum Resistant Ovarian Cancer Cells with an MMP-9/MMP-2 Inhibitor Prior to Cisplatin Enhances Cytotoxicity as Determined by High Content Screening
Source: Int J Mol Sci. 2013 Jan 22;14(1):2085–103. doi: 10.3390/ijms14012085 (PMC3565367; doi:10.3390/ijms14012085)
Supplement: Supplementary file 1 [file ijms-14-02085-s001.pdf]

Supplementary Information

Figure S1. Co-incubation of A2780cis cells with cisplatin and MMP-9/MMP-2i for 6 hours. Simultaneous monitoring of changes in (**A**) cell density, (**B**) cell membrane permeability, (**C**) lysosomal mass/pH, (**D**) nuclear condensation/intensity and (**E**) nuclear size following co-incubation of A2780cis cells with cisplatin and MMP-9/MMP-2i for 6 h. Values were normalised to vehicle treated wells. Representative data are shown as means ± SE ($n = 3$), * $p < 0.05$, ** $p < 0.01$, *** $p < 0.001$. Experiments were carried out in triplicates.

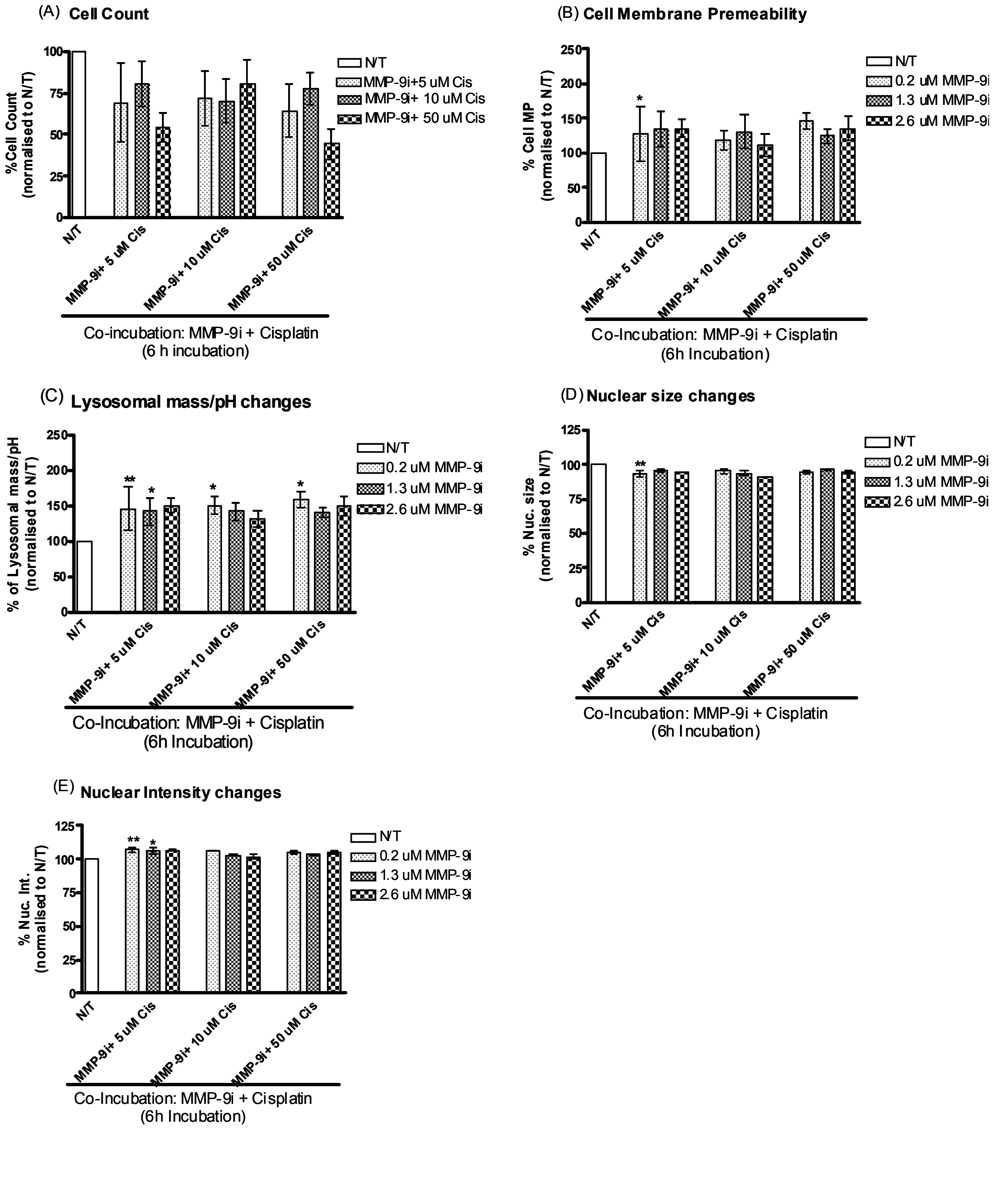

Figure S2. Pre-incubation of A2780cis cells with MMP-9/MMP-2i for 3 h followed by cisplatin for 6 h. Simultaneous monitoring of changes in (**A**) cell density (**B**) cell membrane permeability (**C**) lysosomal mass/pH (**D**) nuclear condensation/intensity and (**E**) nuclear size following pre-incubation of A2780cis cells with MMP-9/MMP-2i for 3 h followed by treatment with cisplatin for 6 hs. Values were normalised to vehicle treated wells. Representative data are shown as means ± SE (n = 3), ** p < 0.01, *** p < 0.001. Experiments were carried out in triplicates.

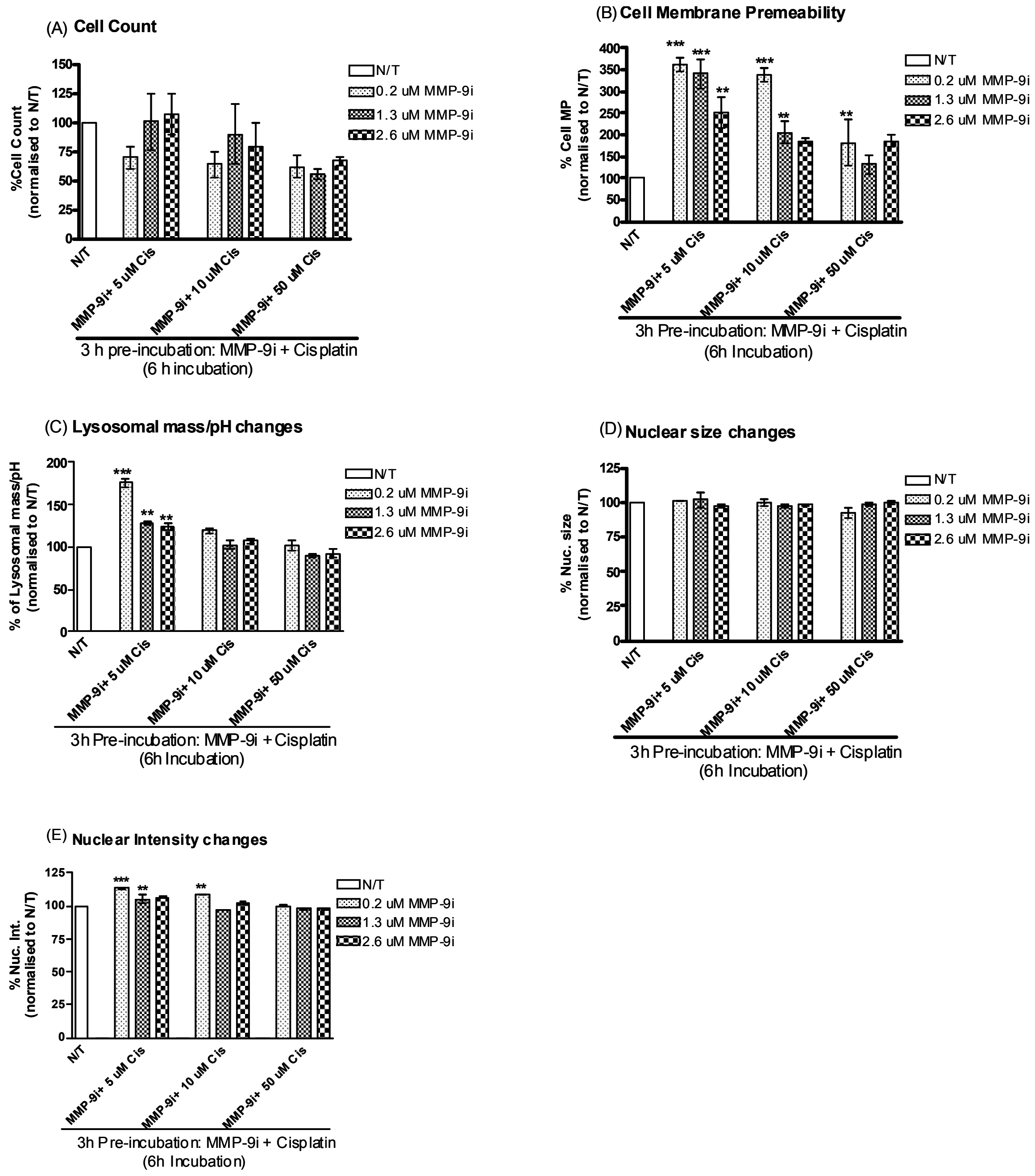

Figure S3. Co-incubation of A2780 cells with cisplatin and MMP-9/MMP-2i. Simultaneous monitoring of changes in (**A**) cell density (**B**) cell membrane permeability (**C**) lysosomal mass/pH (**D**) nuclear condensation/intensity and (**E**) nuclear size following co-incubation of A2780 cells with cisplatin and MMP-9/MMP-2i for 3 h. Values were normalised to vehicle treated wells. Representative data are shown as means ± SE (n = 3), * $p < 0.05$, ** $p < 0.01$, *** $p < 0.001$.

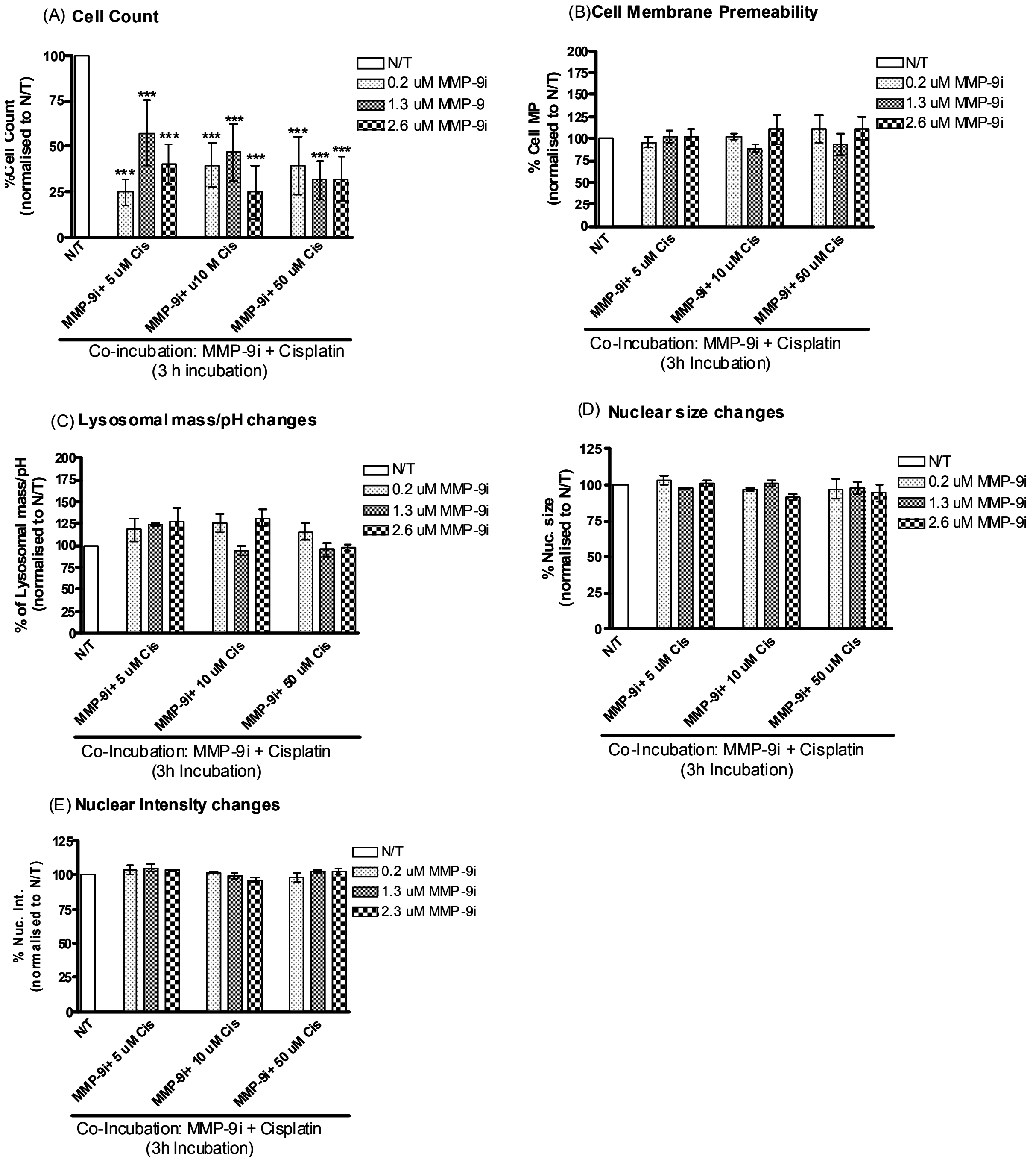

Figure S4. Pre-incubation of A2780 cells with MMP-9/MMP-2i for 3 h followed by cisplatin treatment. Simultaneous monitoring of changes in (**A**) cell density (**B**) cell membrane permeability (**C**) lysosomal mass/pH (**D**) nuclear condensation/intensity and (**E**) nuclear size following pre-incubation of A2780 cells with MMP-9/MMP-2i for 3 h followed by treatment with cisplatin for 3 h. Values were normalised to vehicle treated wells. Representative data are shown as means ± SE ($n = 3$), ** $p < 0.01$, *** $p < 0.001$.

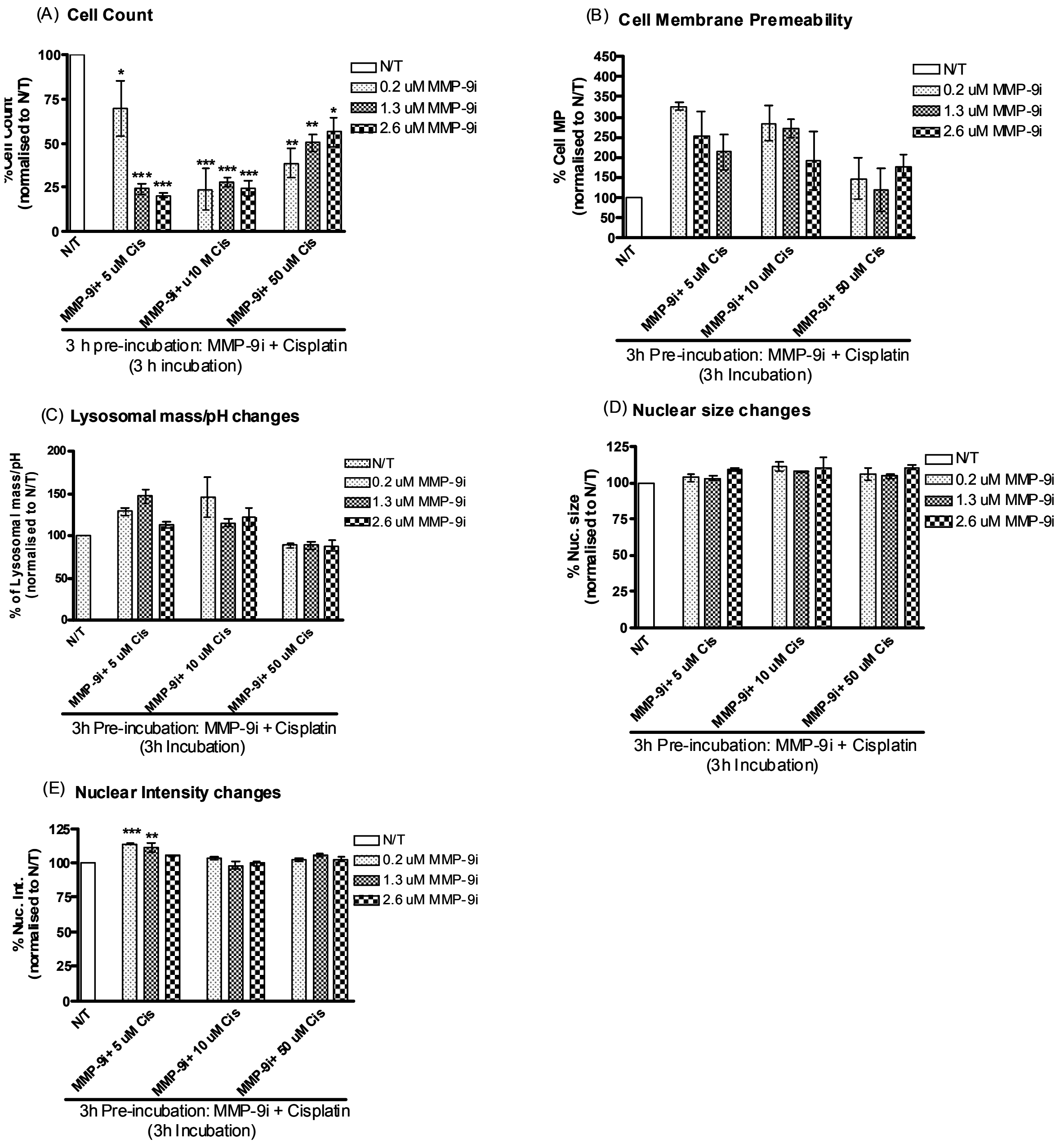

Figure S5. Co-incubation of A2780 cells with cisplatin and MMP-9/MMP-2i for 6 h. Simultaneous monitoring of changes in (**A**) cell density (**B**) cell membrane permeability (**C**) lysosomal mass/pH (**D**) nuclear condensation/intensity and (**E**) nuclear size following co-incubation of A2780 cells with cisplatin and MMP-9/MMP-2i for 6 h. Values were normalised to vehicle treated wells. Representative data are shown as means ± SE ($n = 3$), * $p < 0.05$, ** $p < 0.01$, ***$p < 0.001$. Experiments were carried out in triplicates.

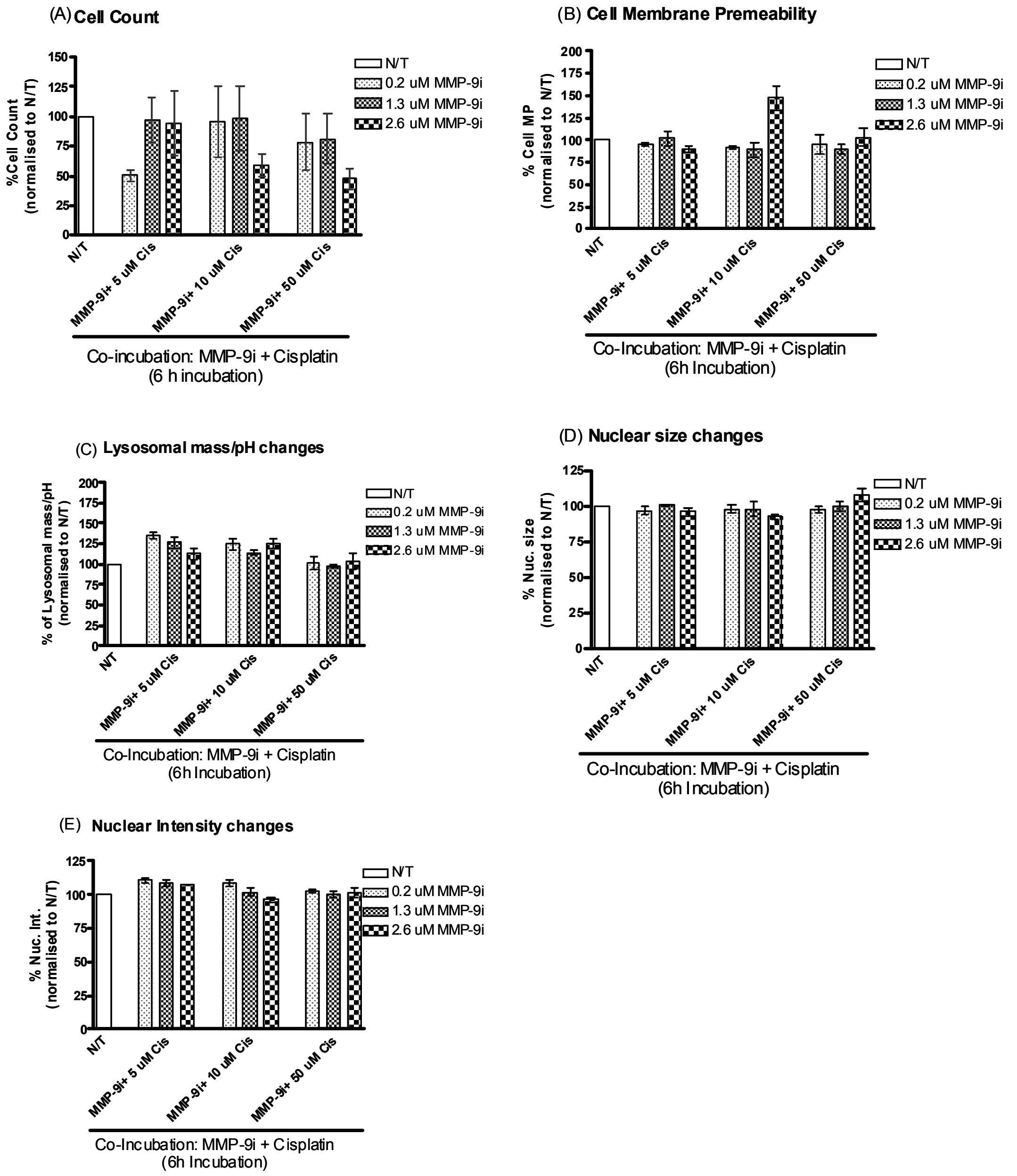

Figure S6. Pre-incubation of A2780 cells with MMP-9/MMP-2i for 3 h followed by cisplatin for 6 h. Simultaneous monitoring of changes in (**A**) cell density (**B**) cell membrane permeability (**C**) lysosomal mass/pH (**D**) nuclear condensation/intensity and (**E**) nuclear size following pre-incubation of A2780 cells with MMP-9/MMP-2i for 3 h followed by treatment with cisplatin for 6 h. Values were normalised to vehicle treated wells. Representative data are shown as means ± SE ($n = 3$), ** $p < 0.01$, *** $p < 0.001$. Experiments were carried out in triplicates.

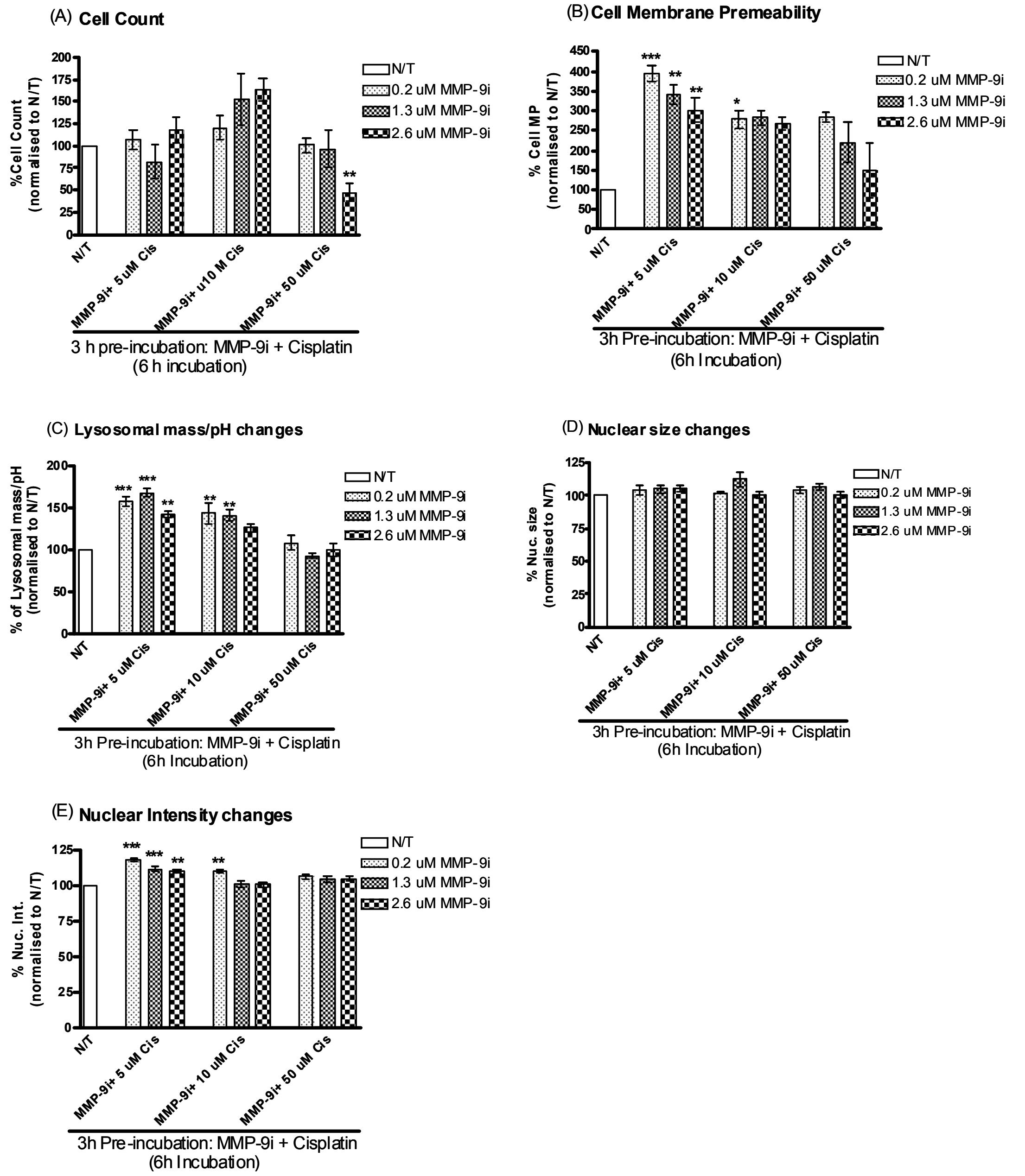

Figure S7. MMP-9 protein expression evaluated by immunohistochemistry in a recurrent serous papillary ovarian adenocarcinoma (20×).

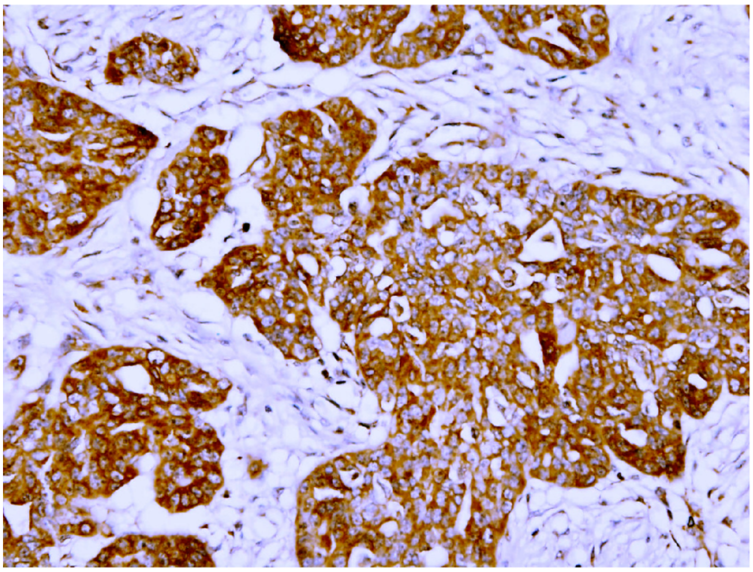

Figure S8. ELISA results of MMP-9 and TIMP-2 concentrations in serum samples. Sample population included serum from benign ovarian cystadenomas, borderline serous ovarian tumours, serous papillary adenocarcinomas of the ovary and recurrent serous papillary adenocarcinomas (*n* = 39).

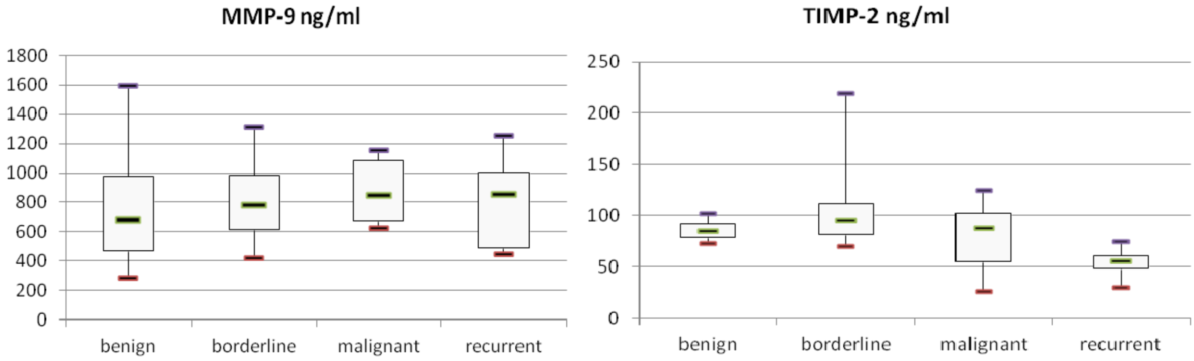

© 2013 by the authors; licensee MDPI, Basel, Switzerland. This article is an open access article distributed under the terms and conditions of the Creative Commons Attribution license (http://creativecommons.org/licenses/by/3.0/).
